# Detection of plasma exosomal miRNA-205 as a biomarker for early diagnosis and an adjuvant indicator of ovarian cancer staging

**DOI:** 10.1186/s13048-022-00961-x

**Published:** 2022-02-19

**Authors:** Zehua Zhu, Zhaojun Chen, Mingxing Wang, Min Zhang, Yiwen Chen, Xiao Yang, Changjun Zhou, Yuhua Liu, Liquan Hong, Lahong Zhang

**Affiliations:** 1 The Medical School of Hangzhou Normal University, Hangzhou, 310015 China; 2 The Clinical laboratory of The Affiliated Hospital of Hangzhou Normal University, Hangzhou, 310015 China; 3 Institute of Basic Medicine and Cancer of The Cancer Hospital of the University of Chinese Academy of Sciences (Zhejiang Cancer Hospital), Hangzhou, 310022 China

**Keywords:** Ovarian cancer, Exosome, miRNA-205, Diagnosis, Biomarker

## Abstract

**Background:**

Ovarian cancer (OC) is one of the serious threats to the health of women worldwide, and accurate biomarkers are urgently demanded for early diagnosis of OC. We have previously confirmed that miR-205 promotes the invasion and metastasis of OC cells by inhibiting the expression of the tumor suppressor gene *TCF21*. In this study, we used liquid biopsy technology to detect the expression levels of the four genes, miR-205, *CA125*, *HE4* and *TCF21*, in the exosomes of plasma of OC patients. Combined with analysis of clinicopathological parameters of OC patients, we aimed to provide efficient and non-invasive laboratory biomarkers for early diagnosis of OC.

**Methods:**

36 OC patients who were diagnosed in local hospitals from September 2020 to July 2021 were selected as OC group, 31 cases of surgically diagnosed with ovarian benign lesions were selected as benign group, and 32 healthy people who underwent physical examination during the same period were selected as a control group. We employed transmission electron microscope (TEM), Western blotting (WB), and nanoparticle tracking analysis (NTA) to identify biomarkers in the exosomes extracted from plasma of the three groups. The RNA levels of miR-205, *CA125*, *HE4* and *TCF2*1 genes in plasma exosomes were detected by real-time quantitative PCR (qRT-PCR) method. We used clinical pathological parameters and the Receiver Operating Characteristic (ROC) curves to evaluate the diagnostic efficacy for the genes detected in plasma exosomes.

**Results:**

We found that the expression level of miR-205 in plasma exosomes of the OC group was significantly higher than that of the benign and control groups (*P* <  0.05), and the level of miR-205 was elevated during the III-IV periods of OC and lymph node metastasis.

**Conclusion:**

The level of miR-205 in plasma exosomes is a valuable tumor biomarker to improve OC diagnosis.

## Background

Ovarian cancer (OC) is one of the most common gynecological malignancies in the female reproductive system with the leading fatality rate, posing a serious threat to the health of women worldwide [1]. Due to the lack of early clinical manifestations and effective screening methods, 70–75% of the OC patients were already at the advanced stage at the time of diagnosis and therefore missed the best treatment opportunity [2]. Thus, OC is called the “silent killer” and there is an urgent need for more efficient, non-invasive, and early screening methods to improve the diagnosis of OC patients.

Liquid biopsy technology represented by detection of biomarkers in extracted exosomes has been reported as a potential tool to solve this problem [3]. Exosomes are tiny vesicles secreted by cells with an unique lipid bilayer membrane structure which well protects the inside contents, such as mRNA and miRNA, from degradation [4]. Therefore, exosomes are much more stable than other biomarkers presented in plasma. In the early stages of tumors, cancer cells secrete a large quantity of exosomes, reaching a level of 10^12^/mL of blood, which makes the quantitative detection of exosomes easier and possible as a new tumor marker [5]. Our previously published studies found that miR-205 is up-regulated in OC tissues. In this study, we mechanistically revealed that miR-205 promoted the invasion and metastasis of OC cells by inhibiting expression of the tumor suppressor gene *TCF21* [6], suggesting that miR-205 has the potential as a novel biomarker for OC diagnosis. Currently, transvaginal ultrasound combined with detection of CA125 and HE4 proteins is mainly used for ovarian cancer screening and diagnosis [7]. However, the detection of CA125 and HE4 have their own limitations in early diagnosis of ovarian cancer, which significantly limits their use in clinical practice [8]. We used the exosomal liquid biopsy technology to detect the expression levels of the four genes, miR-205, *CA125*, *HE4* and *TCF21* in the plasma exosomes of OC patients. Together with analysis of the clinicopathological parameters of OC patients, we explored the diagnostic value and clinical significance of the four genes in plasma exosomes for OC.

## Methods

### Study populations

A total of 99 female subjects were included in the study, including 36 in the OC group, 31 in the benign group, and 32 in the control group. In the OC group, of the 36 ovarian cancer patients, who were admitted to Zhejiang Cancer Hospital from September 2020 to July 2021, 32 had high-grade serous adenocarcinomas, 3 had endometrioid adenocarcinomas, and 1 had clear cell carcinoma. The inclusion criteria were as follows: (1) meeting the relevant diagnostic criteria for OC, and the histological type and the International Union of Obstetrics and Gynecology (FIGO) staging were confirmed by postoperative pathological examination [9]; (2) none of them received any anti-tumor treatments before drawing blood; (3) complete medical records; (4) without other malignant diseases. The benign group had 31 patients with benign ovarian lesions, including 7 serous cystadenoma, 7 endometriotic cyst, 5 mature teratoma, 3 cases of mucinous cystadenoma, 9 cases of simple ovarian cyst. They were surgically diagnosed in the Affiliated Hospital of Hangzhou Normal University during the same period. The control group consisted of 32 healthy women who participated in the physical examination in this hospital during the same period, and excluded for conditions of hypertension, diabetes and diseases or dysfunctions of organs such as heart, liver and kidney. They had no histories of malignant tumors and no dysfunction of important organs.

The OC group had ages ranged from 27 to 90 years with an average of 57 ± 13, the benign group was ranged from 24 to 73 years old with an average of 49 ± 13, and the control group had ages of 27–77 years old with an average of 51 ± 14. There were no significant differences in ages between the three groups (*P* > 0.05). We obtained the informed consent from all individuals, and the study was approved by the hospital ethics committee (Ethics No. 2019 (Len 02)-HS-03).

### Plasma collection

All the subjects without taking foods were drawn 4–6 mL of venous blood anticoagulated with EDTA in the early morning. The blood samples were centrifuged at 3, 500 rpm for 10 min at 4 °C to obtain plasma, which were divided into 2 tubes, one stored at − 80 °C and another one was immediately detected for protein concentrations of CA125 and HE4. If the samples were not detected within 8 h, they were be stored at 4 °C, followed by detection within 24 h.

### Detection of plasma protein CA125 and HE4

The protein concentration of CA125 in plasma was assessed using a Chemiluminescence Immunoassay Analyzer (Abbott ARCHITECT i2000SR, USA), and the HE4 protein concentration using an Electrochemiluminescence Immunoassay Analyzer (Roche Cobas E602, Germany), following the manufacturer’s protocols.

### Exosome isolation

Exosomes were isolated from plasma using Total Exosome Isolation Reagent (#4484450; Invitrogen, USA), according to the manufacturer’s instructions. Briefly, the sample was thawed at room temperature, centrifuged first at 2000 g for 20 min and next at 10,000 g for 20 min to completely remove the cells and debris. Next, 400 μL of the plasma supernatant were mixed with 120 μL of 1 × phosphate buffered saline (PBS, pH 7.4), followed by incubation at 4 °C for 30 min and centrifugation at 10,000 g for 5 min to obtain pellets as exosomes. The exosome samples were stored at − 80 °C for further analysis.

### Transmission electron microscopy (TEM)

Exosomal pellets were resuspend in 1 × PBS, and the suspension was dropped onto the carbon-coated copper mesh for 2 min. After excess liquid was removed, a filter paper was used to drain the grid. Negative staining using phosphotungstic acid was performed for 5 min. The grid was then dried at room temperature, followed by observation at a JEOL-1230 TEM at an acceleration voltage of 100 kV.

### Nanoparticle tracking analysis (NTA)

The size and concentration of the exosomes were determined using NTA (VivaCell Biosceinces) on ZetaView PMX 110 (Particle Metrix, Meerbusch, Germany) and the software ZetaView 8.04.02. Briefly, the ZetaView system was calibrated with 110 nm polystyrene particles, then the separated exosome sample was properly diluted with 1× PBS buffer to measure particle size and concentration. Data were recorded and analyzed using NTA measurements at a room temperature.

### Western blotting analyses (WB)

The exosome samples were treated with lysis buffer to obtain total exosomal protein, which was quantified using BCA method. Lysed exosomal proteins were separated by SDS-PAGE and transferred onto a PVDF membrane. The PVDF membrane was then blocked with BSA at room temperature for 1 h, followed by sequential incubation with primary antibody anti-CD63 or anti-TSG101, overnight and the respective HRP-conjugated secondary antibody. Signals were visualized on a gel imaging system (ChemiDoc XRS, BIO-RAD, USA).

### Extraction of exosomal RNA and reverse transcription

Total RNA was extracted from plasma exosomes using the Multi-type Sample DNA/RNA Extraction-Purification Kit (Sansure Biotech Inc. Hunan, China), following the manufacturer’s instructions. The RNA concentration was measured using the e-spect Spectrophotometer (Beijing labaid science and technology, Ltd., China). The OD 260/280 nm ratios of all RNA samples were ≥ 1.8. A part of the RNA sample were used for reverse-transcription using the Mir-XTM miRNA first-strand synthesis kit (#638313; TAKARA Bio Inc., USA), followed by qRT-PCR for miR-205. The remained RNA sample were stored at − 80 °C and used for qRT-PCR of *CA125*, *HE4* and *TCF21*.

### qPCR analysis for quantification of miR-205

TB Green Advantage qPCR Premix (#639676; TAKARA Bio Inc., USA) was used for qPCR on a 7500 Real-Time PCR System (Applied Biosystems, Thermo Fisher Scientific. Inc., USA). Briefly, two microliters of the cDNA products were used for as PCR template, and the final volume was 25 μl. U6 RNA was used as the internal reference gene, and the reaction conditions were as follows: 95 °C pre-denaturation for 10s, 95 °C denaturation for 5 s, and 60 °C annealing for 20s, a total of 40 cycles to get dissociation curve. The relative expression level of exosomal miR-205 was analyzed using the 2^−ΔΔCt^ relative quantitation method [10].

### qRT-PCR quantification of *CA125*, *HE4* and *TCF21*

The expression mRNA levels of *CA125*, *HE4* and *TCF21* were determined using One Step TB Green®PrimeScript™Plus RT-PCR Kit (#RR096A, TAKARA Bio Inc., USA) on the 7500 Real-Time PCR System. β-actin gene was used as an internal control. The reaction conditions were as follows: reverse transcription of RNA into cDNA at 42 °C for 5 min, 95 °C for 10s, denaturation at 95 °C for 5 s, annealing at 60 °C for 34 s, and a total of 40 cycles. The relative expression of genes was calculated using the 2^-△△CT^ method.

The primers used in this study were purchased from Shanghai Biotech Co., Ltd. China, and the primer sequences are listed in Table 1.

**Table 1:** Primer sequences for gene expression evaluation using qRT-PCR

Genes	Forward Sequence	Reverse Sequence
*CA125*	5 ‘-ACT GCC ACT GAG CCA ACA AGT TC-3’	5 ‘-GAC TGT GCC AAG ACT ATC CGA AGC-3’
*HE4*	5 ‘-TCA ACA GAA GGA GGC AAT GTA T-3’	5 ‘-CAG CTG CTT AAT CTT ATG CTC G-3’
*TCF-21*	5 ‘-CAG CGA TGT GGA GGA CCT TCA AG-3’	5 ‘-TCT CCT CGG TGC TCT CGT TGG-3’
*β-Actin*	5 ‘-CTC CAT CCT GGC CTC GCT GT-3’	5 ‘-GCT GTC ACC TTC ACC GTT CC-3’
*miR-205*	5 ‘-TCCTTC ATT CCA CCG GAG TCT G-3’	MRQ 3′ Primer (provided in the kit)
*U6*	5 ‘-GGA ACG ATA CAG AGA AGA TTA GC-3’	5 ‘-TGG AAC GCT TCA CGA ATT TGC G-3’

### Statistical analysis

All data were analyzed using the SPSS 26.0 statistical software (IBM Corp, USA) and plotted by GraphPad Prism 7.0 (GraphPad Software Inc., USA). All data were expressed as mean ± standard deviation or median (interquartile range), according to data distribution. The Mann-Whitney *U* test was used to compare the two groups, and the Kruskal-Wallis *H* test or one-way ANOVA was used for comparison between multiple groups. The area under the curve (AUC) of the receiver operating characteristic curve (ROC) was used to analyze the diagnostic value of plasma exosomal miR-205, *CA125*, *HE4* and *TCF21* for OC. *P* <  0.05 was considered statistically significant.

## Results

### Characteristics of plasma exosomes

**Fig. 1:**
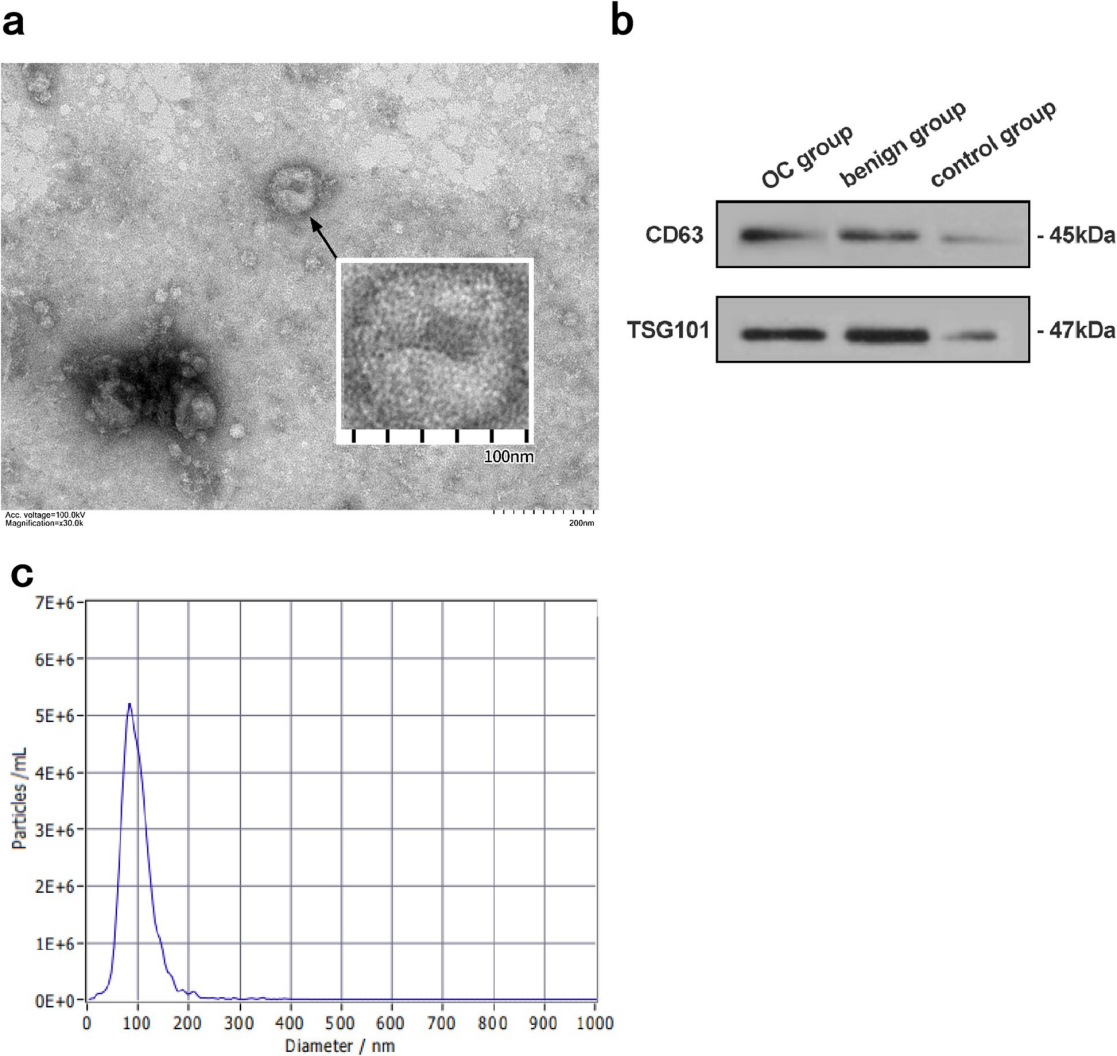
Plasma exosome characteristics. **a** Transmission electron microscopy (TEM) revealed the round shape of exosomes. The bar represents 200 nm. **b**. The marker proteins of exosomes (CD63 and TSG101) were detected by western blotting (WB). **c**. The Nanoparticle tracking analysis (NTA) presents the size and concentration distribution

We analyzed extracted exosomes with by three methods, TEM, NTA and WB. As shown in Fig. 1a, the extracted exosomes observed under TEM were cup-shaped or dish-shaped vesicles with a diameter of about 100 nm**.** WB results showed that the lysates of isolated plasma particles of all groups expressed the exosome-specific marker proteins, CD63 and TSG101 (Fig. 1b). The NTA results showed that the exosomes were distributed as particles with a diameter ranged from 20.7 to 345.2 nm, with an average size of 98.6 nm. And the total concentration of the exosomes was determined at 1.3E+ 12 particles/mL (Fig. 1c).

### Comparison of expression levels of plasma exosomal miR-205, *CA125*, *HE4* and *TCF21* among the three groups of patients and control

**Fig. 2:**
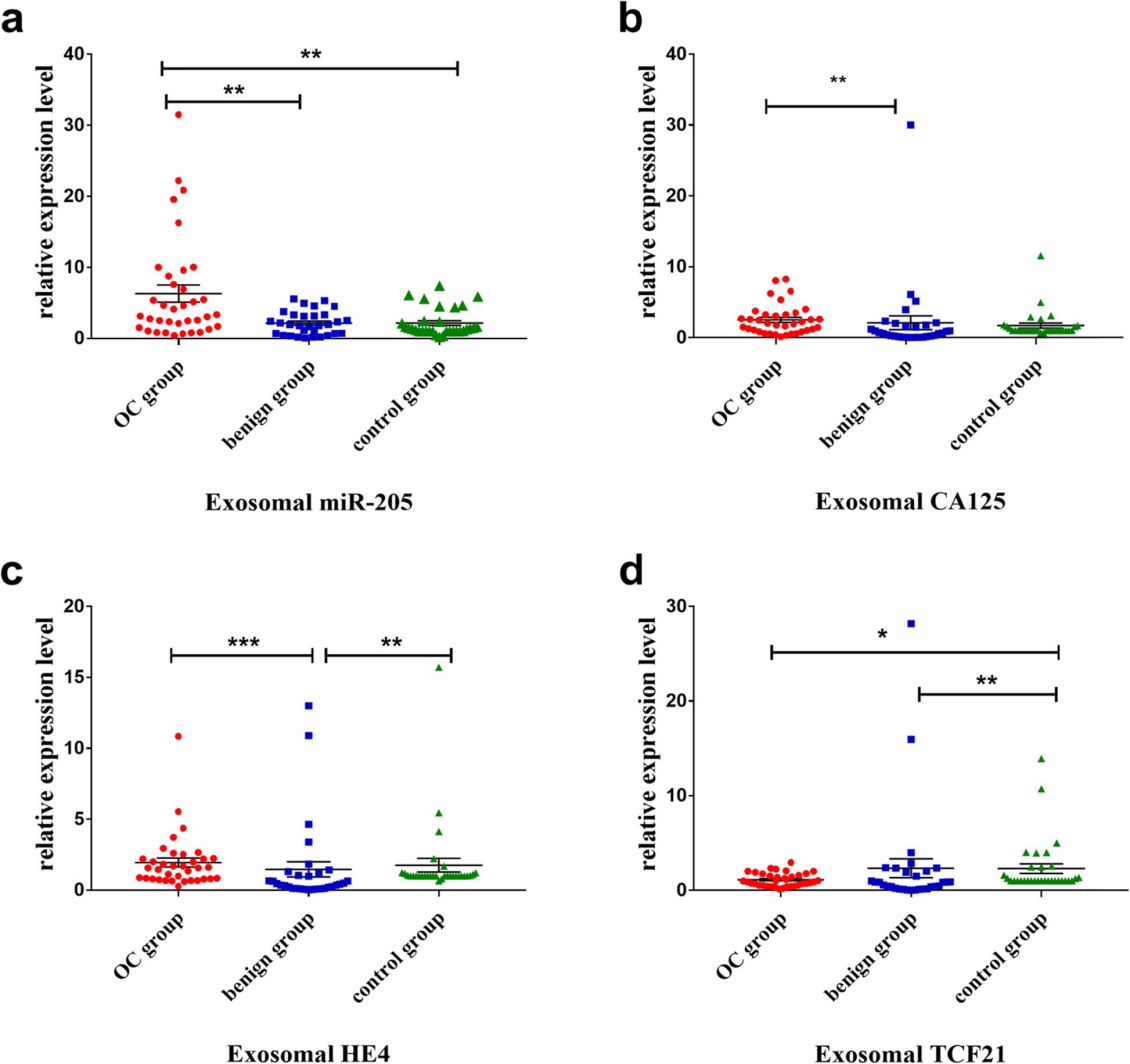
The expression levels of plasma exosomal miR-205, CA125, HE4 and TCF21 among three groups of people (Kruskal-Wallis *H* test). *p < 0.05; **p < 0.01; ***p < 0.001

The expression levels of the four RNAs, *miR-205, CA125, HE4 and TCF21* in the three groups of OC patients and controls were determined by RT-qPCR as described in the **Methods**, and the results are shown in Table 2 and Fig. 2. We found that expression level of plasma exosomal miR-205 in the OC group was significantly higher than those in the benign and control groups (*P* <  0.01) (Fig. 2a). Expression level of plasma exosomes *CA125* in the OC group was the highest among the three groups, which was significantly different from the benign group (*P* <  0.01) (Fig. 2b). The levels of plasma exosomal *HE4* in benign group was significantly lower than those in the OC and control groups (*P* <  0.01) (Fig. 2 c), whereas the expression level of plasma exosomal *TCF21* in the OC group and the benign group was significantly lower than that of the control group (*P* <  0.05) (Fig. 2 d). In addition, there was no significant difference in the expression levels of the four RNAs among the other groups that were not mentioned. (*P* > 0.05).

**Table 2:** Relative expression levels of plasma exosomal miR-205, *CA125*, *HE4* and *TCF21* in OC, benign and control groups [Median(Q1, Q3)]

Tested marker gene	OC group (***n*** = 36)	Benign group (***n*** = 31)	Control group (***n*** = 32)
*miR-205*	3.24 (1.56, 8.48) ^ab^	1.93 (0.67, 3.30)	1.34 (1, 2.41)
*CA125*	1.97 (0.87, 3.25) ^a^	0.66 (0.15, 1.79)	1 (1, 1.56)
*HE4*	1.57 (0.79, 2.23) ^a^	0.42 (0.13, 1.19) ^b^	1 (1, 1.16)
*TCF21*	0.94 (0.51, 1.71) ^b^	0.85 (0.15, 2.04) ^b^	1 (1, 2.47)

^a^indicates that there is a significant difference compared with the benign group (*P* < 0.05)

^b^indicates that there is a significant difference compared with the control group (*P* < 0.05)

*P* values were calculated by the Kruskal-Wallis H test

### The relationship between the levels of the detected genes and the clinicopathological parameters of OC patients

In OC patients, we detected the expression levels of exosomal genes, miR-205, *CA125*, *HE4* and *TCF21* and the protein concentrations of CA125 and HE4 in plasma samples. Together with the analysis of the patient’s clinicopathological parameters, we found that only the expression level of plasma exosomes miR-205 in stage III-IV was higher than that in stage I-II [4.59(2.23,9.39) vs (1.28(0.65, 3.35)]. The level of miR-205 in the group with lymph node metastasis was higher than the group without lymph node metastasis [5.15 (2.40, 10.02) vs 1.69 (0.78, 3.35)], and both were statistically significant (*P* <  0.05). All other analyses suggested there were correlations between the levels of the detected genes in plasma exosomes with age, menopausal status, FIGO stage, lymph node metastasis, and tumor site (*P* > 0.05), as shown in Table 3.

**Table 3:** Relationship between the detected biomarkers and clinicopathological parameters in OC patients

Tested biomarkers	Age (< 50 years vs. ≥ 50 years)	Menopause (Yes vs. No)	Metastases lymph nodes (Yes vs. No)	FIGO stage (stage I + II vs. stage III + IV)	Tumor site (unilateral vs. bilateral
	*P value*				
exosomal *miR-205*	0.689	0.768	0.007*	0.032*	0.553
exosomal *CA125*	0.849	0.664	0.292	0.845	0.761
exosomal *HE4*	0.689	0.520	0.435	0.192	0.665
exosomal *TCF21*	0.337	0.286	0.588	0.557	0.911
protein CA125(U/mL)	0.614	0.271	0.830	0.614	0.665
protein HE4 (pmol/L)	0.374	0.286	0.934	0.362	0.170

*P* values were calculated by Mann-Whitney *U* test. * *P* < 0.05

### Evaluate the diagnostic value of the four genes and protein CA125 and HE4 of plasma exosomes in OC

**Fig. 3:**
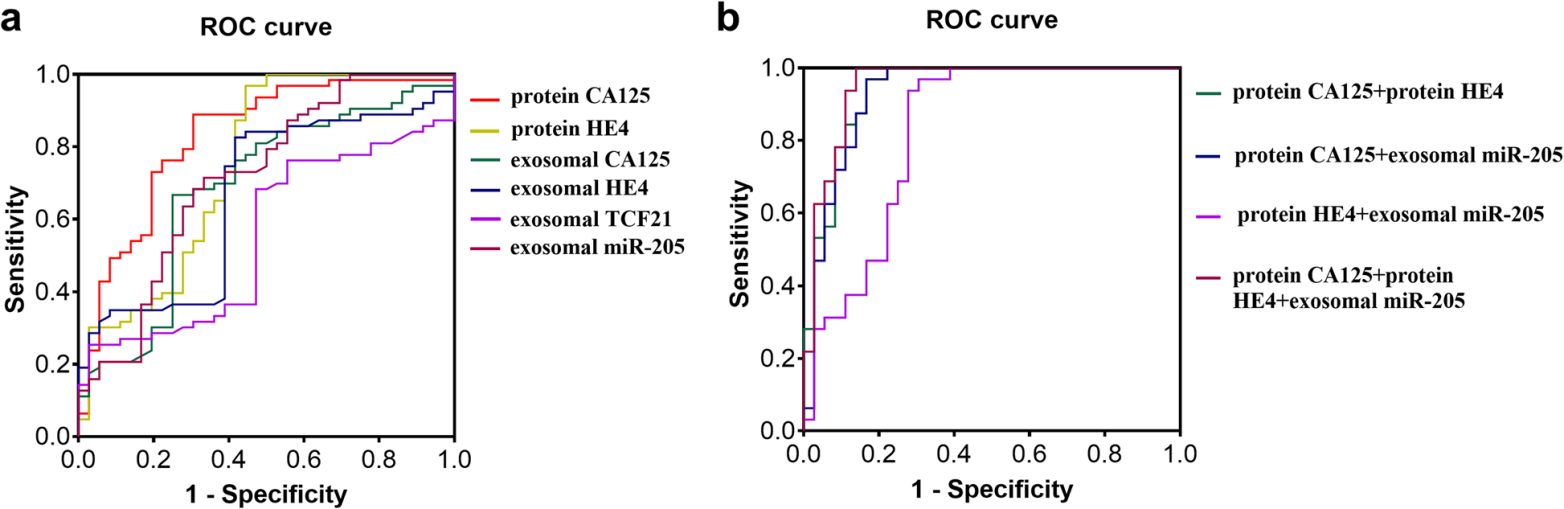
Receiver operator characteristics (ROC) curves for prediction of OC by the levels of plasma exosomal miR-205 and *CA125*, *HE4* and *TCF21* mRNAs alone or combined with traditional biomarkers, CA125 and HE4 proteins

ROC curves were used to further evaluate the diagnostic efficacy of plasma exosomal genes miR-205, *CA125*, *HE4*, *TCF21* and plasma exsosomal proteins CA125 and HE4 concentration for OC, using the control group as a reference (Table 4 and Fig. 3**)**. The AUC of plasma exosomal miR-205 was 0.715 (95% *CI*: 0.590–0.841, *P* = 0.002), with a sensitivity of 66.7% and a specificity of 78.1%. The AUC of plasma exosomal *CA125*, *HE4*, and *TCF21* were 0.642, 0.554, and 0.673, respectively, which were all less than 0.7, suggesting they are not ideal for diagnosis of OC. The AUC of protein CA125 was 0.915 (95% *CI*: 0.846–0.983, *P* <  0.001), with a sensitivity of 74.3% and a specificity of 93.7%, and the AUC of protein HE4 was 0.779 (95%*CI*: 0.668–0.889, *P* < 0.001), with a sensitivity of 55.6% and a specificity of 100%. The combined index analysis showed that exosomal miR-205 together with protein CA125 or HE4, the diagnostic AUC was 0.930 (95% CI: 0.865–0.995, *P* < 0.0001) or 0.827 (95% CI: 0.726–0.929, *P* < 0.0001). The AUC of the triple gene diagnosis was increased to 0.951 (95%CI: 0.899–1.004, *P* < 0.0001), and the sensitivity and the specificity was increased to 100 and 86.1%, respectively. However, the AUC of traditional plasma proteins, CA125 and HE4, was 0.939 (95% CI: 0.883–0.995, *P* < 0.0001), the sensitivity was 96.9%, and the specificity was 83.3%.

**Table 4:** Diagnostic value of single detected biomarker and combined detections for OC

Test biomarkers	AUC	95% CI	***P***	Sensitivity%	Specificity%	Youden’s index
protein CA125 (U/mL)	0.915	0.846–0.983	< 0.001	74.3	93.7	0.68
protein HE4 (pmol/L)	0.779	0.668–0.889	< 0.001	55.6	100.0	0.56
exosomal miR205	0.715	0.590–0.841	0.002	66.7	78.1	0.45
exosomal *CA125*	0.642	0.501–0.784	0.044	75.0	68.7	0.44
exosomal *HE4*	0.554	0.403–0.704	0.446	58.3	84.4	0.43
exosomal *TCF21*	0.673	0.539–0.808	0.014	47.2	59.4	0.07
protein CA125 + protein HE4	0.939	0.883–0.995	< 0.0001	96.9	83.3	0.80
protein CA125 + exosomal miR-205	0.930	0.865–0.995	< 0.0001	96.9	83.3	0.80
protein HE4 + exosomal miR-205	0.827	0.726–0.929	< 0.0001	96.9	69.4	0.66
protein CA125 + protein HE4 + exosomal miR-205	0.951	0.899–1.004	< 0.0001	100	86.1	0.86

## Discussion

OC is the fifth most common tumor that threatens the health of women worldwide, and its mortality rate ranks the first among gynecological tumors [11]. According to the latest cancer report in 2021, the incidence of OC accounts for about 3.4% of all tumors, but the mortality rate is nearly 5%, which means that there are approximately 310,000 new cases and 200,000 deaths in 2020 [1]. Early detection and early diagnosis are the key to improving the prognosis of OC patients. However, studies have shown that the conventional screening method of serum CA125 combined with vaginal ultrasound cannot reduce the mortality of patients [12, 13]. At present, there are no effective screening methods for OC, and the diagnosis must rely on the pathological results of tissue biopsy, which brings great pain to patients [14]. The exosomes-based liquid biopsy technology can probe disease progressions through non-invasive or minimally invasive sampling. In recent years, it has received more and more attention in tumor screening, diagnosis and treatment, and prognosis evaluation [15]. This also provides new ideas for OC screening and early diagnosis.

In this study, we extracted exosomes in plasma samples of all subjects with commercial reagents, and then used three methods (TEM, WB, and NTA) to analyze the extracted exosomes. They were visually observed under TEM. The particles were in the form of cup-shaped vesicles with a diameter of about 100 nm. WB results showed that the particles expressed exosomal characteristic proteins CD63 and TSG101. NTA results showed that the average size of the particles was 98.6 nm and contained a high concentration (1.3E+ 12 particles/mL). These results were consistent with the characteristics of exosomes as previously reported [16], and confirmed the success in extraction of plasma exosomes. Exosomes are involved in the communication between cells, and they are widely present in biological cells and various body fluids [17]. Exosomes derived from cancer cells in the early stages of tumors can be released into the blood in large quantities and are highly stable in peripheral blood, making it possible to be used as new tumor markers. A number of studies have shown that exosomes play an important role in the occurrence, development and drug resistance of ovarian cancer, and therefore exosomes have the capability to guide the diagnosis, treatment and prognosis evaluation of OC [18–21].

We further analyzed the expression levels of the four genes *miR-205*, *CA125*, *HE4* and *TCF21* in plasma exosomes and the concentrations of plasma proteins, CA125 and HE4. We found that the expression level of plasma exosomal miR-205 in the OC group were significantly higher than that in the benign and control groups, which was consistent with our previous results in OC tissues and cell lines [6]. At the same time, together with the analysis of the clinicopathological parameters of OC patients, we found that the expression level of plasma exosomal miR-205 in OC patients was higher in advanced-stage (stage III-IV) than in early-stage (stage I-II), the group with lymph node metastasis was higher than the group without lymph node metastasis, and the differences were statistically significant. However, the traditional serum markers of plasma proteins, CA125 and HE4, were not related to the FIGO staging of OC and lymph node metastasis. Compared with traditional markers, plasma exosomal miR-205 performs better in early diagnosis and prognostic evaluation of OC. Thus, it is expected that exosomal miR-205 will become a novel tumor biomarker to assist in the screening and early diagnosis of OC. We previously have shown that miR-205 was highly expressed in OC tissues, and miR-205 mimic in cell lines promotes the invasion and metastasis of OC cells. We highly suspect that the high expression of miR-205 in OC tissues may transmit relevant signals through exosomes, thereby accelerating the overall invasion and metastasis process of OC. All these indicate that plasma exosomal miR-205 becomes an early diagnostic biomarker for OC and can be used for prognostic evaluation of OC.

MiR-205 is one of the highly conservative miRNAs. It has a dual effect as both a tumor promotion factor and a tumor suppressor [22]. Studies have found that the expression of miR-205 was reduced in sera of breast cancer patients [23], but it was up-regulated in OC tissues and cells and related to the growth and metastasis of OC [24], which was consistent with our previous published results [6]. In addition, it has been suggested that vascular endothelial growth factor (VEGF) upregulates miR-205 expression, which leads to the downregulation of Ezrin and Lamin A/C and, thereby, promotes the invasion of OC cells [25]. miR-205 has been shown to regulate the proliferation and invasion of OC cells by inhibiting the expression of PTEN/Smad4 [26]. Other studies have shown that miR-205 directly negatively regulates ZEB1 to promote the clinical progress of EOC patients [27]. A recent study also showed that miR-205 derived from OC cell exosomes promoted the transformation of OC by inducing angiogenesis [28]. Therefore, together with the results obtained from our current study, these studies confirmed that miR-205 had abnormal expression in OC and was related to the invasion of OC.

We further evaluated the diagnostic value of plasma exosomal miR-205 for OC, together with the current traditional biomarkers, plasma proteins CA125 and HE4. Through ROC curve analysis, it was found that the AUC of plasma proteins CA125 and HE4 were 0.915 and 0.779, respectively, the sensitivity was 74.3 and 55.6%, and the specificity was 93.7 and 100%, respectively. This result confirmed that although the traditional biomarkers provide valuable diagnosis of OC, they are less sensitive, resulting in missing diagnoses. The plasma exosomal miR-205 alone diagnosed OC with an AUC of 0.715, a sensitivity of 66.7%, and a specificity of 78.1%. However, when combined with the two traditional markers, plasma exosomal miR-205 significantly improved the early diagnosis of OC, which increased the AUC of CA125 from 0.915 to 0.930, and HE4 from 0.779 to 0.827. Moreover, combination of the detection of the three biomarkers increases the AUC to 0.951, the sensitivity to 100%, and the specificity to 86.1%. Taken together, plasma exosomal miR-205 expression is of a high value in OC diagnoses, and when combined with the traditional biomarkers, CA125 and HE4 proteins, it effectively improves the diagnostic effectiveness of OC. Importantly, our study supports the detection of all the three biomarkers are, in particular, suitable for early screening of OC.

The study also found that the plasma exosomal *CA125* and *HE4* mRNA levels in the OC group were higher than those in the benign group, but there was no statistical difference from the control group. It was inconsistent with the findings by Fawzy et al. that the expression levels of *CA125* and *HE4* in OC tissues were higher than those of benign tumor tissues and normal ovarian tissues [29]. In addition, the expression level of plasma exosomal *TCF21* in the OC and benign groups was lower than the control group, but there was no difference between the OC and benign groups. This was inconsistent with our previous results found in OC tissues [6]. The reason for this inconsistency is likely due to the inherent differences in gene expression patterns in plasma exosomes and tissues. Studies have shown that the differential expression profile of serum exosomal mRNA was not completely consistent with the tissue mRNA level [30], which explains the inconsistency to some points. On the other hands, it may also be due to the selective packaging of the mRNA molecules contained in exosomes, as exosomes regulate the packaging process of their internal RNA through a variety of ways [31]. In addition, the effects of differences in mRNA stability, sample source, sample size, and storage time may also account for the variation.

Although our study demonstrates the advantages of the use of plasma exosomal miR-205 as a new biomarker for OC, it has some limitations, for example, the small samples, which may lead to variations. In future studies, we will increase the sample size, preferably including cases from multiple hospitals. Nevertheless, the expression of miRNA had tissue and disease specificity [32, 33], and can be secreted in the form of exosomes and stably exists in the peripheral blood circulation [34]. It can also be delivered to the tumor microenvironment or distant organs through exosomes, and promotes tumor angiogenesis and metastasis by targeting gene expression in recipient cells [35]. We conclude that plasma exosomal miR-205 is a novel biomarker for OC, which is of a high reference value for early screening of OC.

## Conclusions

In summary, our results showed that plasma exosomal miR-205 has an obvious merit in OC diagnosis, and when combined with the traditional serological tumor biomarkers, it improves the diagnostic efficiency of OC. In addition, the plasma exosomal miR-205 level is related to OC staging and lymph node metastasis, which provides a valued reference for the early diagnosis and prognostic evaluation of OC patients.

## Data Availability

The data supporting this study are all included in this article.

## Abbreviations

OC: Ovarian cancer
CA125: Cancer antigen 125
FIGO: International Federation of Gynecology and Obstetrics
HE4: Human epididymis protein 4
ROC: Receiver operating characteristic curves
AUC: Area under the curve
CI: Confidence interval
TCF21: Transcription factor 21
